# Utility of next generation sequencing in paediatric neurological disorders: experience from South Africa

**DOI:** 10.1038/s41431-024-01582-2

**Published:** 2024-05-03

**Authors:** Magriet van Niekerk, Shahida Moosa, Ronald van Toorn, Regan Solomons

**Affiliations:** 1 Department of Paediatrics and Child Health, Faculty of Medicine and Health Sciences, Stellenbosch University, Cape Town, South Africa; 2 Division of Molecular Biology and Human Genetics, Faculty of Medicine and Health Sciences, Stellenbosch University, Cape Town, South Africa; 3 Medical Genetics, Tygerberg Hospital, Cape Town, South Africa

**Keywords:** Epilepsy, Diseases

## Abstract

Next generation sequencing (NGS)-based tests have become routine first-line investigative modalities in paediatric neurology clinics in many high-income countries (HICs). Studies from these countries show that these tests are both cost-effective and reliable in diagnosing many complex childhood neurological diseases. However, NGS-based testing in low-and middle-income countries (LMICs) is limited due to affordability constraints. The primary objective of this study was to evaluate the diagnostic yield and impact of targeted gene panel sequencing in a selected paediatric cohort attending a tertiary paediatric neurology clinic in the Western Cape Province of South Africa. This retrospective study included 124 consecutive paediatric patients with neurological disease, aged 6 weeks to 17 years, referred for NGS-based multi-gene panel testing over a 41-month period. Twenty-four different disease group-specific panels were utilized. A caregiver experience questionnaire was administered when a pathogenic variant was identified. The overall study diagnostic yield (DY) was 45% (56/124 patients). The diagnostic yield in this study is similar to previously reported paediatric cohorts in HICs. The high yields for neuromuscular disorders (52%) and early epileptic encephalopathies (41%) suggest that NGS-based panels may be more cost-effective as first-line testing in well-defined phenotypes. The latter finding argues for early inclusion of all children with developmental epileptic encephalopathies (DEE), as early diagnosis leads to better treatment and avoidance of unnecessary investigations.

## Introduction

The rapidly expanding field of paediatric neurogenetics has transformed our understanding of mechanisms mediating neurological diseases. Precise and timeous diagnosis of these diseases are essential to be able to offer appropriate treatment and family counselling. Studies have shown that extensive non-genetic investigations of childhood neurological diseases (epileptic encephalopathies, movement disorders, neuromuscular disorders, and developmental delay, amongst others) are often costly with low diagnostic yields, resulting in diagnostic failure or delay [1].

In 2011, the World Health Organization (WHO) recommended the implementation of community genetics programmes in LMICs [2] The aim of the programmes was the reduction of congenital disorders and genetic diseases at the population level, in addition to providing genetic services, including diagnosis and counselling, for individuals and families. Implementation challenges identified by the WHO include the uneven distribution of diagnostic genetic laboratories (603 worldwide, 256 outside the USA and only 20 in LMICs) as well as cost constraints [3] Encouraging findings include that the cost of NGS-based testing is expected to decrease 30-fold within several years, whilst DNA collection via dried blood spots, buccal swabs and saliva offer an attractive solution for sample acquisition in LMICs, given the cost, ease of collection and transportation of samples. Unaffordability is often raised as an insurmountable obstacle in LMICs. However, this notion only considers the costs of testing and not the cost of the disease burden. Paradoxically, LMICs are likely to benefit the most from NGS-based testing due to the lack of screening, higher rates of consanguinity and higher infant mortality rates (3) [3]. The WHO also reported that reconfiguration of sequence capacity to allow testing for the most relevant and potentially treatable diseases encountered in LMICs might also bring about further cost reduction [2].

Southern Africa as a region offers rich human genetic diversity, which has relevance when studying and informing diagnostic testing that is universally applicable. Genetics data from LMICs should be included when NGS panels are configured and variants are interpreted. This study aimed to evaluate the impact of genetic testing and the diagnostic utility of targeted gene panel sequencing in a selected paediatric cohort attending a tertiary paediatric neurology clinic in the Western Cape Province of South Africa.

## Materials and methods

### Participants

We retrospectively collected data from 124 consecutive paediatric patients with neurological disease aged 6 months to 17 years, referred over a 41-month period to the tertiary paediatric neurology outpatient service at Tygerberg Hospital, which is situated in the Western Cape Province of South Africa. This hospital serves a population of approximately 2.6 million people, which includes an estimated paediatric population of 1,062,911 children under the age of 13 years. Close to 2000 children are seen annually in the paediatric neurology outpatient service of the hospital. Most families reside in poor underserviced communities. A paediatric neurologist and/or developmental paediatrician and medical geneticist examined all children. Clinical data analysed included age of onset of the disease, neurological symptoms, presence of developmental delay, neurology examination findings and the results of all requested special investigations. Locally available genetic testing was preferentially offered via the National Health Laboratory Service (NHLS); however, these tests are unfortunately not comprehensive. Some of these routinely available tests include Spinal Muscular Atrophy (SMA) (*SMN* homozygous deletion exon 7 only), Becker and Duchenne Muscular Dystrophy (BMD, DMD) (deletions, duplications only), *RYR1* (founder variants only), Charcot-Mary-Tooth 1A (CMT1A) (*PMP2* duplication only) and Myotonic Dystrophy (Triplet repeat, Polymerase Chain Reaction (PCR)). Data from these tests are not included here. Exome sequencing is only available on a research basis, as costs are not covered by the hospital.

### Sample and data collection

Following pre-test genetic counselling and informed consent, buccal swabs, assisted saliva or blood samples (3 ml) were collected from all the patients. The sampling method depended on the patient’s age, clinical condition, and ability to provide a saliva sample.

### Next-generation sequencing-based gene panels

NGS-based gene panels were outsourced (Invitae, USA). A list of panels and genes included are available in Supplementary Table 1. The panel selected depended on the suspected underlying neurological disorder. In total, twenty-four panels were used in the study (Supplementary Tables 2 and 3), covering most of the disease groups presenting to paediatric neurology. The test results were categorized as either: positive (pathogenic/likely pathogenic variant identified), inconclusive (variant(s) of uncertain significance (VUS) identified), or negative (no variants identified).

### Recommended treatment and opportunities for precision medicine

Where possible we collected information on the current therapy and which changes were made after a genetic diagnosis was confirmed and the opportunity to institute precision care was available. This was not part of the original study and had to be done retrospectively therefore the data is unfortunately limited.

### Qualitative questionnaire

Subsequently, six questions relating to caregiver experience were administered when a pathogenic variant was identified (Supplementary Table 4).

### Comparison to other NGS studies

We did an internet search for related studies. We then included these studies in Supplementary Table 9 for comparison. The studies we used met the following criteria: they were published in the last 10 years, used NGS panels, participants had paediatric neurological conditions.

### Statistical analysis

Data were analysed using the statistical software SPSS version 26.0. We calculated the diagnostic yield by dividing the number of patients with a positive result over the total number of tests performed. The data obtained from the questionnaire was categorical (yes/no/some/unknown/not applicable). The variables were summarized using frequency and proportions. The study was approved by the Stellenbosch University Health Research Ethics Committee, HREC Reference No: S22/03/034.

## Results

### Patient characteristics

Overall, 124 patients underwent gene panel testing. One-hundred and twenty-seven panels were utilized: for patient 43 a broader panel was requested after the original smaller panel was negative, for patient 51 a customised panel was requested to include further genes, and for patient 86 a combined Epilepsy and Cerebral Palsy Spectrum Disorders panel was requested.

The median age of the patient at the time of gene panel analysis was 63 months (interquartile range (IQR): 12–96 months). Fifty (40%) of patients were tested by 2 years of age and 77 (62%) by 5 years of age. There was a 1.1:1 ratio of female to 6 male patients. The median specimen turnaround time was 6 weeks (IQR: 4–8 weeks).

### Pathogenic variants identified and diagnostic yield

The overall study diagnostic yield (DY) was 45% (56/124 patients) (Supplementary Table 5). Supplementary Table 2 illustrates the 70 pathogenic variants identified in the study, by panel and individual gene. Supplementary Table 3 shows the disorder groups that were tested, the number of panels requested, as well as the diagnostic yield for each panel and group. Diagnostic yields were highest for neuromuscular disorders 52% (13/25) followed by epilepsies 41% (26/63 patients) and cerebral palsy spectrum disorders 31% (5/16 patients). The small number of patients in some phenotypic groups limited the interpretation of specific diagnostic yields. A total of 48 patients (39%) had at least one VUS.

### Gene-phenotype matching

Supplementary Table 5 shows details of the patients in whom a diagnosis could be made definitively, along with the pathogenic variants identified. 17 children with developmental epileptic encephalopathies (DDE) from a previous study were included in Supplementary Table 5, as they were all tested within the same time frame [4]. A total of 46% (26/56) of patients had an autosomal dominant, 30% an autosomal recessive (17/56 - 10/17 homozygous and 7/17 compound heterozygous) and 23% (13/56) an X-linked condition.

Parental/family testing was undertaken for 38 patients. This was mainly for VUS resolution (13/38). For 21 patients, the mother was available for testing, for 2 patients only the father was available and for 13 both parents were available. In a further 2 cases, parents and a sibling were tested and confirmed the diagnosis of autosomal dominant *ADAR*-related dyschromatosis symmetrica hereditaria (mother and brother of Patient 68) and confirmed the absence of the pathogenic variant in *ARSA* in the younger brother of Patient 60. Testing of parental samples were helpful for confirming de novo status of the variants (7 patients) and phasing of variants (8 patients). Further information on 5 patients in whom only 1 pathogenic variant was identified in a recessively inherited gene can be found in Supplementary Table 6. The second variant in the respective gene was not identified, thus the patients were only confirmed to be heterozygous carriers and a confirmatory diagnosis could not be made despite a high clinical suspicion. In a further 7 patients, pathogenic variants were identified which were not considered clinically relevant or contributing to the presenting phenotype. For example, patient 96 was found to be a carrier of a pathogenic heterozygous variant in *ATM*, related to increased susceptibility to autosomal-dominant breast cancer, which was not related to the current phenotype, however, the gene was included because in the homozygous/compound heterozygous state, variants in *ATM* cause ataxia telangiectasia. Further incidental findings not considered to be contributing to the presenting phenotypes are presented in Supplementary Table 7.

In this cohort, we identified the same variant in at least 2 patients for the following genes:

· *ARSA* c.905 G > A, p.Cys302Tyr was identified in Patient 60 (homozygous) and in Patient 92 (heterozygous) - known pathogenic variant in ClinVar (VCV000963270.8)

· *CDKL5* c.1927C>T, p.Gln643* was identified in Patient 77 and Patient DEE 2 (hemizygous) - no submissions in ClinVar (VCV001070908.2)

· *RYR1* c.10348-6 C > G was identified in Patients 21 (heterozygous), Patient 62 (heterozygous) and Patient 67 (heterozygous) - known pathogenic variant in ClinVar (VCV000132994.40)

· *STAC3* c. 851 G > C, p.Trp284Ser was identified in Patient 58 (homozygous) and Patient 107 (homozygous) - known pathogenic variant in ClinVar (VCV000088744.48)

Further interesting case reports are available in Supplementary Document 1 (Interesting cases).

### Opportunities for precision medicine

Although this was not part of the original study an attempt was made to look at a subset of patients, where we would potentially have been able to change treatment and optimise care according to the genetic diagnosis (Supplementary Table 5). A total of 31 of the 56 patients would have benefitted from some form of precision medicine. In 11/31 of these patients the specific therapy is not yet available in our setting. The subgroup that received the most benefit was the epilepsy group as medication avoidance or add-on therapy is relatively easy to do, for example, Patient DEE9 and DEE12 (Supplementary Table 5).

## Utility to parents

Supplementary Table 8 illustrates the caregiver responses to positive (pathogenic) test results. Twenty-eight parents (97%) felt that knowing the result brought closure for the family and 28 (97%), indicated that should prenatal testing be available that they would use it in future pregnancies. In 14 (48%) of the cases, treatment was adjusted to some extent once the pathogenic variant was known. Fourteen parents (48%) felt that it assisted in helping them take better care of their child (Supplementary Table 8).

## Discussion

This study aimed to evaluate the diagnostic yield and the impact of targeted gene panel sequencing in a selected cohort attending a LMIC tertiary paediatric neurology outpatient service. Due to the location of the OPD in an LMIC, the bulk of the pathology seen at the paediatric neurology OPD is cerebral palsy (mostly secondary to hypoxic ischaemia) and neurological sequelae secondary to infective causes like bacterial and tuberculous meningitis. Therefore, although the numbers of patients seen at the OPD is large, the number of eligible participants for this study was limited.

The overall study diagnostic yield (DY) was 45% (56/124 patients). It was highest for neuromuscular disorders 52% (13/25) followed by epilepsies 41% (26/63) and cerebral palsy spectrum disorders 31% (5/16). Of the neuromuscular disorders, the two largest groups were congenital myopathies 44% (7/16) and muscular dystrophies 31% (5/16).

The patients with cerebral palsy were a heterogenous group of patients with spasticity or dystonia or a mixed spastic-dystonia. They had no birth or past medical history to explain their clinical picture. They all had brain imaging where structural causes for the cerebral palsy were ruled out.

No exome sequencing testing was included in this study as this is a very costly resource, the cost of which is currently not covered by the hospital. The results point to the fact that although some candidate genes may be missed when exomes are not used, the yield of NGS panels when applied in this clinical setting still has much to contribute to diagnosing paediatric neurology patients.

To date, several studies have investigated the diagnostic yield of molecular testing in paediatric neurological disorders. The overall rate of molecular diagnosis in previous studies range from 20 to 60%. However, the results of previous studies are not generalizable, due to heterogeneous study designs, sample size, inclusion criteria, and specific diagnostic techniques. To the best of our knowledge, this is the first study that specifically investigated the diagnostic yield of commercially available gene panels in a large cohort attending a tertiary paediatric neurology outpatient service in an LMIC. The overall diagnostic yield in this study is similar to previously reported paediatric cohorts in HICs ([5–16]; Supplementary Table 9).

Clinical interpretation of genetic test results is increasingly complicated by variants of uncertain significance (VUS) that have an unknown impact on health. High volumes of returned VUS (48/124 patients) in the study similarly complicated interpretation of results. This is true across disease category and across panels, as our population is understudied and underrepresented in global databases [17]. Reclassification can clarify a variant’s clinical significance and it is increasingly facilitated by the availability of updated information about human genetic diversity, especially among underrepresented populations. This furthermore highlights the importance of studies, which include LMICs and diverse populations.

The most common genes identified in the study were *SCN1A* (*n* = 7), *CDKL5* (*n* = 4), *DMD* (*n* = 4), *KCNQ2* (*n* = 3), *RYR1* (*n* = 3). These genes have been well described in other international paediatric studies. *SCN1A, CDKLA5* and *KCNQ2* were also among the top five genes with the highest yield using NGS in studies focusing on paediatric epilepsy in a study by Mei et al. [18]. A recent study from the South Africa using a panel of 71 DEE-associated genes identified Pathogenic/Likely pathogenic candidate single nucleotide variant or short indels in 12% of cases (28/234 patients) [19]. Similar to our study, *SCN1A* proved the most prevalent gene in this category (*n* = 13). Of interest was the failure to identify any *KCNQ2* cases despite the gene’s inclusion in the testing panel. The three *KCNQ2* cases identified in our study highlight the importance of including this gene in any DEE or Epilepsy panel, as it is relevant and potentially allows clinicians to offer precision therapy. Precision therapy in *KCNQ2* varies depending on whether the variant is gain of function (GoF) or loss of function (LoF). In GoF cases, the suggested therapy is Sodium Channel Blockers while in LoF Retigabine is advised [20].

Although precision therapy may have proven benefit, it is not always available in all countries. We have indicated some of the known therapies and whether they are available in our Hospital in Supplementary Table 5. The International League against epilepsy has written a report on the availability and affordability of antiseizure medication (ASM) in different countries [20]. This report highlights the need for an uninterrupted supply of at least three ASM on the WHO essential drug list in each country, price regulations of ASM, and to increase the in-country capacity for production of essential medication [21, 22]. In our experience, there is a discrepancy in what is available in LMIC and High-Income Countries (HIC) as well as between state and public sector within the same country. For example, Cannabidiols and Biotin are more accessible in our private sector than in our public sector hospitals. Also, some medications in the public sector are limited to only tertiary institutions, like Clobazam and Vigabatrin. This means that patients need to travel far and at great cost to get the medication every month which leads to poor adherence and increase in seizure frequency.

Spinal muscular atrophy is a commonly encountered neuromuscular disease in our region. Studies have shown that the birth incidence of SMA in black South Africans is higher than one in 3574 [23]. Local testing for *SMN1* does not include a determination of *SMN2* copy numbers. The latter may be employed to correlate with the disease phenotype, predict disease evolution, and stratify patients that are eligible for gene therapy. The Invitae SMA panel used in this study offered the additional advantage of identifying the *SMN2* copy numbers, facilitating easier entry into clinical trials for these patients.

Even in this relatively small study, a few recurrent pathogenic variants have been identified in unrelated patients. For example, the *STAC3* c.851 G > C, p.Trp284Ser has since been confirmed in a further two unrelated isiXhosa speaking South African patients seen in our service. This is the first report of STAC3-related myopathy from Southern Africa. This will enable us to establish local testing for this variant, as we have previously done for other founder variants. The *CDKL5* c.1927C > T, p.Gln643* pathogenic variant identified in 2 patients has not been previously described.

### Utility to the parents

Parents of patients with positive results were given the opportunity to partake in this study by voluntarily answering a six-question questionnaire (Supplementary Table 4). The majority of parents felt that knowing the result brought closure and that if prenatal testing should be available, they would make use of it in future pregnancies. In almost half of the cases, treatment was adjusted to some extent once the pathogenic variant was known. Treatment changes included adjusting anti-seizure medications e.g., stopping sodium channel blockers like Lamotrigine in patients with *SCN1A* or adding sodium channel blockers like carbamazepine in patients with *KCNQ2* pathogenic variants [4]. The questionnaire was given to parents only and not the treating physicians therefore full details of treatment changes were not covered by this study.

### Strengths and limitations

The study was unique to a specific population of patients from a single centre. VUS resolution was not performed on all patients, as either one (especially the father) or both biological parents were not available to provide samples. As this was a gene panel testing approach, the analysis was limited to the genes in the panel and the diagnostic assays used. For the genes included in the panel, certain types of variants may be missed (intronic, structural, some deletions/duplications). In addition, cost limitations prohibited more detailed genetic testing (for example, exome or genome for patients with a negative result) or further functional analysis (for example, RNA sequencing or in-vitro ion channel analysis for patients with VUSs in ion-channel genes). Some patients with inconclusive results have been enrolled in further research.

Despite these limitations, our study provides insights into the diagnostic yield of NGS in a resource-constrained, previously non-investigated, LMIC setting with high levels of genetic diversity. Testing allowed clinicians to optimise genetic counselling, patient care and prognostication when pathogenic variants were identified. Caregivers were able to receive closure and make plans for their families.

## Conclusion

The study enforces that NGS testing is achievable in resource-constrained settings. The high diagnostic yield in this study suggests that it is feasible to recommend NGS as a first-tier testing approach for children with neurological disorders.

## Supplementary information

Supplementary Table 1.

Supplementary Table 2

Supplementary Table 3.

Supplementary Table 4

Supplementary Table 5

Supplementary Table 6

Supplementary Table 7

Supplementary Table 8

Supplementary Table 9

## Data Availability

All data generated are available in this article and supplementary files. Testing was conducted at Invitae (USA) and variants have been submitted to ClinVar as per Invitae practice.
