# Supplementary material for: Utility of next generation sequencing in paediatric neurological disorders: experience from South Africa
Source: Eur J Hum Genet. 2024 May 3;32(10):1314–8. doi: 10.1038/s41431-024-01582-2 (PMC11499987; doi:10.1038/s41431-024-01582-2)
Supplement: Supplementary file 1 — Supplementary Table 1. [file 41431_2024_1582_MOESM1_ESM.docx]

**Supplementary Table 1. The number of genes in each panel.**

|  | **Panel** | **Genes tested in each panel** | **Number of genes** |
| --- | --- | --- | --- |
| 1. | Epilepsy panel | *AARS, ABAT, ADAR, ADSL, ALDH5A1, ALDH7A1, ALG1, ALG12, ALG13, ALG6, AMACR, AMT, AP2M1, AP3B2, ARG1, ARHGEF9, ARSA, ARX, ASAH1, ASNS, ATAD1, ATP1A1, ATP1A2, ATP1A3, ATP6AP2, ATP7A, ATRX, BRAT1, C12ORF57, CACNA1A, CACNA1B, CACNA1E, CACNA2D2, CAD, CAMK2B, CARS2, CASK, CCDC88A, CDKL5, CHD2, CHRNA2, CHRNA4, CHRNB2, CLCN4, CLCN6, CLN2 (TPP1), CLN3, CLN5, CLN6, CLN8, CLTC, CNTN2, CNTNAP2, COG5, COL18A1, CSTB, CTNNB1, CTSD, CYFIP2, CYP27A1, DDC, DDX3X, DEAF1, DENND5A, DEPDC5, DHDDS, DHFR, DIAPH1, DMXL2, DNAJC5, DNM1, DNM1L, DOCK7, DYNC1H1, DYRK1A, ECHS1, EEF1A2, EHMT1, EMC1, EPM2A, FAR1, FARS2, FBXO11, FGF12, FLNA, FOLR1, FOXG1, FOXP1, FRRS1L, GABBR2, GABRA1, GABRA2, GABRB1, GABRB2, GABRB3, GABRG2, GAMT, GATAD2B, GATM, GCH1, GLDC, GLRA1, GLRB, GNAO1, GNB1, GOSR2, GPAA1, GPHN, GRIA3, GRIN1, GRIN2A, GRIN2B, GRIN2D, GTPBP3, HCN1, HDAC8, HEXA, HNRNPU, IER3IP1, IFIH1, IQSEC2, ITPA, KANSL1, KCNA1, KCNA2, KCNB1, KCNC1, KCND2, KCNH1, KCNH2, KCNH5, KCNJ10, KCNK4, KCNMA1, KCNQ2, KCNQ3, KCNQ5, KCNT1, KCTD7, KIF1A, KIF2A, KIF5A, KPNA7, LAMC3, LGI1, LIAS, MBD5, MDH2, MECP2, MEF2C, MFSD8, MICAL1, MOCS1, MOCS2, MTOR, NACC1, NAGLU, NECAP1, NEDD4L, NEXMIF, NGLY1, NHLRC1, NPC1, NPC2, NPRL3, NRXN1, NTRK2, NUS1, PACS1, PACS2, PAFAH1B1, PCDH19, PCLO, PEX10, PEX12, PEX13, PEX14, PEX16, PEX19, PEX2, PEX26, PEX3, PEX5, PEX6, PHGDH, PIGA, PIGB, PIGG, PIGN, PIGO, PIGP, PIGQ, PIGV, PIGW, PLAA, PLCB1, PNKD, PNKP, PNPO, PNPT1, POLG, PPP2CA, PPP2R1A, PPP2R5D, PPP3CA, PPT1, PRICKLE1, PRIMA1, PROSC, PRRT2, PSAP, PSAT1, PSPH, PTEN, PTPN23, PURA, QARS, QDPR, RAB11A, RAB11B, RAI1, RALA, RANBP2, RELN, RFT1, RHOBTB2, RNASEH2A, RNASEH2B, RNASEH2C, RNF13, ROGDI, RORB, RUSC2, SAMHD1, SATB2, SCARB2, SCN1A, SCN1B, SCN2A, SCN3A, SCN8A, SCN9A, SCP2, SERPINI1, SETBP1, SGCE, SGSH, SIK1, SLC12A5, SLC13A5, SLC19A3, SLC1A2, SLC25A12, SLC25A22, SLC2A1, SLC35A2, SLC6A1, SLC6A5, SLC6A8, SLC9A6, SMC1A, SNAP25, SNX27, SPATA5, SPTAN1, ST3GAL3, ST3GAL5, STAG2, STRADA, STX1B, STXBP1, STXBP2, SUMF1, SUOX, SURF1, SYN1, SYNGAP1, SYNJ1, SZT2, TANGO2, TBC1D24, TBCK, TBL1XR1, TCF4, TH, TK2, TPK1, TREX1, TRIM8, TSC1, TSC2, TSFM, TUBB2A, UBA5, UBE3A, UNC80, WDR45, WWOX, YWHAG, ZDHHC9, ZEB2, ZSWIM6* | 302 |
| 2. | Comprehensive neuromuscular panel | *BHD5, ACAD9, ACADM, ACADVL, ACTA1, ADSSL1, AGK, AGL, AGRN, AHCY, ALDOA, ALG14, ALG2, AMACR, AMPD1, ANO5, ATP2A1, ATP7B, B3GALNT2, B4GAT1, BAG3, BIN1, C1QBP, CACNA1S, CAPN3, CASQ1, CAV3, CCDC78, CFL2, CHAT, CHKB, CHRNA1, CHRNB1, CHRND, CHRNE, CLCN1, CNTN1, COL12A1, COL13A1, COL6A1, COL6A2, COL6A3, COLQ, COQ2, COQ4, COQ7, COQ8A, COQ9, COX15, COX20, COX6B1, CPT1A, CPT2, CRYAB, CTDP1, DAG1, DDC, DES, DGUOK, DMD, DNA2, DNAJB6, DNM2, DOK7, DPAGT1, DPM1, DPM2, DPM3, DYSF, EMD, ENO3, ETFA, ETFB, ETFDH, FBXL4, FDX2, FHL1, FKBP14, FKRP, FKTN, FLAD1, FLNC, GAA, GATM, GBE1, GFER, GFPT1, GMPPB, GNE, GOSR2, GYG1, GYS1, HACD1, HADH, HADHA, HADHB, HMBS, HNRNPA2B1, HNRNPDL, ISCU, ISPD, ITGA7, KBTBD13, KCNJ2, KLHL40, KLHL41, LAMA2, LAMP2, LARGE1, LDB3, LDHA, LMNA, LMOD3, LPIN1, MAN2B1, MAP3K20, MATR3, MEGF10, MGME1, MICU1, MPV17, MTM1, MUSK, MYH2, MYH3, MYH7, MYL2, MYO18B, MYOT, MYPN, NEB, OPA1, OPA3, ORAI1, PDSS1, PDSS2, PFKM, PGAM2, PGK1, PGM1, PHKA1, PHKB, PLEC, PNPLA2, PNPLA8, POGLUT1, POLG, POLG2, POMGNT1, POMGNT2, POMK, POMT1, POMT2, PREPL, PUS1, PYGM, PYROXD1, RAPSN, RBCK1, RNASEH1, RRM2B, RXYLT1, RYR1, SCN4A, SDHA, SELENON, SGCA, SGCB, SGCD, SGCG, SIL1, SLC16A1, SLC18A3, SLC22A5, SLC25A20, SLC25A3, SLC25A4, SLC25A42, SLC5A7, SMCHD1, SMN1, SMN2, SMPX, SPEG, SQSTM1, STAC3, STIM1, SUCLA2, SUCLG1, SYT2, TANGO2, TAZ, TCAP, TIA1, TK2, TNNT1, TNPO3, TOR1AIP1, TPM2, TPM3, TRAPPC11, TRIM32, TRMT5, TSFM, TTN, TWNK, TYMP, VAMP1, VCP, VMA21, YARS2* | 211 |
| 3. | Early infantile epileptic encephalopathy panel | *ALDH7A1, ARHGEF9, ARX, BRAT1, CACNA2D2, CASK, CDKL5, CHD2, CLCN4, DNM1, DOCK7, EEF1A2, FARS2, FOLR1, FRRS1L, GABBR2, GABRA1, GABRB3, GNAO1, GRIN1, GRIN2A, GRIN2B, HCN1, HNRNPU, IER3IP1, KCNA2, KCNB1, KCNMA1, KCNQ2, KCNQ3, KCNT1, PCDH19, PIGA, PIGN, PIGO, PLCB1, PNKP, PNPO, PURA, SCN1A, SCN2A, SCN8A, SCN9A, SIK1, SLC12A5, SLC13A5, SLC25A12, SLC25A22, SLC2A1, SLC35A2, SLC6A1, SMC1A, SPTAN1, STXBP1, SYNGAP1, SZT2, TBC1D24, WDR45, WWOX, ARHGEF15, ATP1A2,*  *COQ4, GPHN, KCNH5, MTOR, NECAP1, NEDD4L, SCN1B, ST3GAL3* | 69 |
| 4. | Comprehensive neuropathies panel | *AARS, AIFM1, APOA1, ASAH1, ATL1, ATL3, ATP1A1, ATP7A, BAG3, BICD2, BSCL2, CHCHD10, COX6A1, CYP27A1, CYP7B1, DCTN1, DHTKD1, DNAJB2, DNM2, DNMT1, DRP2, DST, DYNC1H1, EGR2, ELP1, EXOSC9, FBLN5, FBXO38, FGD4, FIG4, GAN, GARS, GDAP1, GJB1, GLA, GNB4, GSN, HARS, HEXA, HINT1, HMBS, HSPB1, HSPB8, IGHMBP2, INF2, KIF1A, KIF5A, LITAF, LMNA, LRSAM1, MARS, MCM3AP, MED25, MFN2, MME, MORC2, MPZ, MTMR2, NDRG1, NEFH, NEFL, NGF, NTRK1, PDK3, PLEKHG5, PMP2, PMP22, POLG, POLG2, PRDM12, PRPS1, PRX, RAB7A, REEP1, RETREG1, SBF1, SBF2, SCN11A, SCN9A, SEPT9, SH3TC2, SIGMAR1, SLC12A6, SLC25A46, SLC52A2, SLC52A3, SLC5A7, SMN1, SMN2, SPG11, SPTLC1, SPTLC2, SURF1, TFG, TRIM2, TRPV4, TTR, UBA1, VAPB, VRK1, WNK1, YARS, ARHGEF10, CCT5, HSPB3, LAS1L, MICAL1, SCN10A, SGPL1, SLC25A21, SLC52A1* | 111 |
| 5. | Hereditary sensory and autonomic neuropathy panel | *ATL1, ATL3, DNMT1, DST, ELP1, KIF1A, NGF, NTRK1, PRDM12, RETREG1, SCN11A, SCN9A, SPTLC1, SPTLC2, WNK1* | 15 |
| 6. | Leukodystrophy and leukoencephalopathy panel | *AARS, AARS2, ABAT, ABCA1, ABCD1, ABCD4, ABHD5, ACADS, ACBD5, ACER3, ACO2, ACOX1, ACP5, ACSF3, ACTA2, ACTB, ACY1, ADAR, ADGRG1, ADK, ADNP, ADSL, AGA, AHDC1, AHI1, AIFM1, AIMP1, AIMP2, ALDH18A1, ALDH3A2, ALDH5A1, ALDH6A1, ALG12, ALG13, ALG2, ALG6, ALG9, AMACR, AMPD2, AMT, ANK3, AP1S2, AP3B2, AP4B1, AP4E1, AP4M1, AP4S1, AP5Z1, APC2, APOPT1, ARCN1, ARFGEF2, ARHGAP31, ARHGEF9, ARNT2, ARSA, ARX, ASL, ASNS, ASPA, ASS1, ASXL1, ASXL2, ATP13A2, ATP5A1, ATP6AP2, ATP6V1A, ATP7A, ATP7B, ATP8A2, ATPAF2, ATRN, ATRX, AUH, B3GALNT2, B4GALNT1, BCAP31, BCAT2, BCKDHA, BCKDHB, BCL11B, BCS1L, BICD2, BMP4, BOLA3, BPTF, BRAT1, BTD, C12ORF57, C12ORF65, C19ORF12, C2CD3, CACNA1A, CACNA1E, CARS2, CBS, CC2D2A, CCDC88A, CDC42, CDKL5, CEP290, CHMP1A, CLCN2, CLCN4, CLCN7, CLN2 (TPP1), CLN3, CLN5, CLN6, CLN8, CLP1, CLPP, CNNM2, CNOT1, CNTNAP1, CNTNAP2, COA7, COASY, COG7, COL18A1, COL3A1, COL4A1, COL4A2, COLGALT1, COQ2, COQ6, COQ7, COQ8A, COQ9, COX10, COX14, COX15, COX20, COX6B1, COX7B, COX8A, CPLANE1, CPLX1, CPS1, CRAT, CREBBP, CRIPT, CRLF1, CSF1R, CSPP1, CTBP1, CTC1, CTDP1, CTNS, CTSA, CTSD, CUL4B, CYB5R3, CYFIP2, CYP27A1, CYP2U1, CYP7B1, D2HGDH, DAG1, DARS, DARS2, DBT, DCAF17, DCX, DDC, DDHD1, DDHD2, DDOST, DDX3X, DEAF1, DEGS1, DGUOK, DHCR24, DHFR, DHX37, DLD, DLL4, DMXL2, DNM1L, DNM2, DOCK6, DOCK7, DOLK, DONSON, DPAGT1, DPM1, DPM2, DPM3, DPYS, DYRK1A, EARS2, EDNRB, EHMT1, EIF2AK1, EIF2AK2, EIF2B1, EIF2B2, EIF2B3, EIF2B4, EIF2B5, ELOVL1, ELOVL4, ENTPD1, EOGT, EPG5, EPRS, ERCC1, ERCC2, ERCC3, ERCC6, ERCC8, ETFA, ETFB, ETFDH, ETHE1, EXOSC2, EXOSC3, EXOSC8, EXOSC9, FA2H, FAM126A, FAR1, FARS2, FARSB, FASTKD2, FBXL4, FDX2, FGFRL1, FH, FIG4, FKRP, FKTN, FLVCR2, FOLR1, FOXC1, FOXG1, FOXRED1, FUCA1, FUK, GAA, GABBR2, GABRA1, GABRB1, GABRB2, GABRB3, GALC, GALT, GAN, GATAD2B, GCDH, GDAP1, GFAP, GFM1, GFM2, GJA1, GJB1, GJC2, GLA, GLB1, GLDC, GLRX5, GLUL, GLYCTK, GM2A, GMNN, GNAO1, GNS, GOT2, GPHN, GRIN1, GRM7, GTF2H5, GTPBP2, HACE1, HCN1, HEPACAM, HERC1, HEXA, HEXB, HIBCH, HIKESHI, HK1, HLCS, HMGCL, HNRNPU, HSD17B10, HSD17B4, HSPD1, IARS, IARS2, IBA57, IDH2, IDS, IDUA, IER3IP1, IFIH1, INPP5E, IREB2, ISCA1, ISCA2, ITPA, ITPR1, IVD, JAM3, KARS, KAT6B, KATNB1, KCNJ10, KCNJ2, KCNMA1, KCNT1, KCTD7, KDM1A, KIAA0556, KIAA0586, KIAA0753, KIDINS220, KIF1A, KIF1C, KLHL7, KMT2E, L2HGDH, LAGE3, LAMA1, LAMA2, LAMB1, LARGE1, LARS2, LETM1, LIAS, LIPT1, LIPT2, LONP1, LRPPRC, LYRM7, MAG, MAGEL2, MAN2B1, MANBA, MARS2, MAST1, MAT1A, MCCC1, MCCC2, MCOLN1, MDH2, MECP2, MED17, MED25, MEF2C, MFSD2A, MFSD8, MGP, MICU1, MKS1, MLC1, MLYCD, MMACHC, MMADHC, MOCS1, MOCS2, MOGS, MORC2, MPLKIP, MPV17, MPZ, MRPL12, MRPL44, MRPS16, MRPS22, MRPS34, MSTO1, MTFMT, MTHFR, MTHFS, MTO1, MTOR, MTR, MTRR, MUT, NACC1, NADK2, NAGA, NAGLU, NAGS, NANS, NARS2, NAXD, NAXE, NDRG1, NDUFA1, NDUFA10, NDUFA11, NDUFA12, NDUFA13, NDUFA2, NDUFA4, NDUFA6, NDUFA9, NDUFAF1, NDUFAF2, NDUFAF3, NDUFAF4, NDUFAF5, NDUFAF6, NDUFB10, NDUFB11, NDUFB3, NDUFB8, NDUFB9, NDUFS1, NDUFS2, NDUFS3, NDUFS4, NDUFS6, NDUFS7, NDUFS8, NDUFV1, NDUFV2, NEK1, NFE2L2, NFU1, NGLY1, NKX6-2, NOTCH1, NPC1, NPC2, NPHP1, NRXN1, NSUN3, NT5C2, NTRK2, NUBPL, NUP62, OAT, OCRL, OPA1, OPA3, OSGEP, OTC, PACS1, PAFAH1B1, PAH, PANK2, PARS2, PAX1, PC, PCCA, PCCB, PCDH12, PCGF2, PDGFRB, PDHA1, PDHB, PDHX, PDP1, PET100, PEX1, PEX10, PEX11B, PEX12, PEX13, PEX14, PEX16, PEX19, PEX2, PEX26, PEX3, PEX5, PEX6, PEX7, PGAP1, PGK1, PHGDH, PHYH, PIGA, PIGB, PIGG, PIGL, PIGM, PIGN, PIGP, PIGQ, PIGT, PIGU, PIGV, PIK3C2A, PIK3R2, PLA2G6, PLAA, PLEKHG2, PLP1, PMM2, PMP22, PNKP, PNPT1, POLG, POLG2, POLR1A, POLR1C, POLR3A, POLR3B, POMGNT1, POMK, POMT1, POMT2, PPP1R15B, PPP2R1A, PPP3CA, PPT1, PRF1, PRKDC, PRODH, PROSC, PRPS1, PRUNE1, PSAP, PSAT1, PSPH, PTEN, PTPN23, PURA, PUS3, PYCR2, QARS, QRSL1, RAB11B, RAB3GAP1, RAB3GAP2, RARS, RARS2, RBPJ, REPS1, RERE, RHOBTB2, RIN2, RMND1, RNASEH2A, RNASEH2B, RNASEH2C, RNASET2, RPGRIP1L, RPIA, RPS6KC1, RRM2B, RTTN, RXYLT1, SAMD9L, SAMHD1, SCN3A, SCO1, SCO2, SDHA, SDHAF1, SDHB, SDHD, SEPSECS, SERAC1, SETBP1, SGSH, SH3TC2, SHOC2, SHPK, SLC12A2, SLC12A5, SLC12A6, SLC13A3, SLC13A5, SLC16A2, SLC17A5, SLC19A3, SLC1A2, SLC1A3, SLC1A4, SLC25A1, SLC25A12, SLC25A15, SLC25A19, SLC25A22, SLC25A4, SLC25A42, SLC25A46, SLC2A1, SLC30A10, SLC33A1, SLC35A2, SLC39A14, SLC39A8, SLC46A1, SLC6A19, SLC6A8, SLC6A9, SLC9A1, SLC9A6, SMC1A, SNAP29, SNIP1, SNORD118, SNRPB, SNX14, SOD1, SON, SOX10, SOX2, SPART, SPAST, SPATA5, SPG11, SPG7, SPTAN1, SQSTM1, SRD5A3, SSR4, ST3GAL5, STAG2, STAMBP, STAT1, STAT2, STN1, STRADA, STX11, STXBP1, STXBP2, SUCLA2, SUCLG1, SUMF1, SUOX, SURF1, SYNE1, SYNJ1, TACO1, TAF2, TANGO2, TARS2, TBC1D24, TBCD, TBCE, TBCK, TBX1, TCF4, TCTN2, TIMM50, TIMMDC1, TM4SF20, TMEM106B, TMEM126B, TMEM165, TMEM216, TMEM67, TMEM70, TMTC3, TOE1, TP53RK, TPI1, TPK1, TRAPPC11, TRAPPC9, TREX1, TRMT10A, TRMT5, TSC1, TSC2, TSEN54, TTC19, TUBA1A, TUBA8, TUBB2A, TUBB2B, TUBB3, TUBB4A, TUBG1, TUFM, TWNK, TXN2, TYMP, UBA5, UBE2A, UBE3A, UFM1, UNC13D, UPB1, USP7, VARS2, VPS11, VPS13D, VPS33A, WARS2, WDR45, WDR73, WHSC1, WWOX, YARS, YME1L1, ZEB2, ZFYVE26, ZIC1, ZNF335* | 697 |
| 7. | Metachromatic leukoencephalopathy panel | *ARSA, PSAP, SUMF1* | 3 |
| 8. | Cerebral palsy spectrum disorders panel | *ABAT, ABCD1, ACADM, ACADVL, ACAT1, ACBD5, ACOX1, ACTB, ADAR, ADCY5, ADD3, ADNP, ADSL, AFG3L2, AGAP1, AHDC1, AHI1, AKT3, ALDH18A1, ALDH3A2, ALDH5A1, ALDH7A1, ALG13, ALG3, ALS2, AMACR, AMPD2, AMT, ANO3, AP4B1, AP4E1, AP4M1, AP4S1, AP5Z1, APTX, ARG1, ARHGEF9, ARL6IP1, ARSA, ARX, ASL, ASNS, ASPA, ASS1, ASXL1, ATAD1, ATL1, ATM, ATP13A2, ATP1A3, ATP7A, ATP7B, ATP8A2, ATRX, AUH, AUTS2, B4GALNT1, BCAP31, BCKDHA, BCKDHB, BICD2, BSCL2, BTD, C12ORF65, C19ORF12, CACNA1A, CACNA1G, CAMTA1, CAPN1, CASK, CBS, CCDC88C, CCT5, CDKL5, CEP290, CHD8, CHRNA1, CIZ1, CLN2 (TPP1), CLN3, CLN5, CLN6, CLN8, CLTC, COASY, COL4A1, COL4A2, COL6A3, COQ2, COQ4, COQ6, COQ7, COQ8A, COQ9, CPS1, CPT1C, CREBBP, CTBP1, CTNNB1, CTSD, CYP27A1, CYP2U1, CYP7B1, DARS, DARS2, DBH, DBT, DCAF17, DDC, DDHD1, DDHD2, DDX3X, DGKZ, DHDDS, DHFR, DLAT, DLD, DMD, DNAJC12, DNM2, DPAGT1, DYNC1H1, DYRK1A, EEF2, EHMT1, EIF2B1, EIF2B2, EIF2B3, EIF2B4, EIF2B5, ELOVL4, ELOVL5, ENTPD1, EPHA4, ERCC6, ERCC8, ERLIN1, ERLIN2, ETFA, ETFB, ETFDH, ETHE1, EXOSC3, FA2H, FAM126A, FARS2, FAT2, FGF12, FGF14, FH, FOLR1, FOXG1, FRRS1L, FTL, FUCA1, GABRA2, GAD1, GALC, GAMT, GATM, GBA, GBA2, GCDH, GCH1, GFAP, GJC2, GLB1, GLDC, GLRA1, GLRB, GM2A, GNAL, GNAO1, GNB1, GNS, GPHN, GPR88, GRID2, GRIN1, GRIN2B, GRM1, HACE1, HESX1, HEXA, HEXB, HGSNAT, HLCS, HMGCL, HPCA, HPRT1, HSD17B10, HSD17B4, HSPD1, IBA57, IFIH1, IQSEC2, IREB2, ITPA, ITPR1, KANK1, KAT6A, KCNA2, KCNC3, KCNJ6, KCNMA1, KCNQ2, KCNT1, KCTD17, KCTD7, KDM5C, KIDINS220, KIF1A, KIF1C, KIF5A, KMT2A, KMT2B, KMT2C, L1CAM, L2HGDH, LAMA2, LIAS, LMBRD1, MAG, MAOA, MAP2K1, MARS2, MAST1, MCCC1, MCCC2, MCEE, MECP2, MECR, MFSD8, MICU1, MLYCD, MMAA, MMAB, MMACHC, MMADHC, MOCS1, MOCS2, MOCS3, MPC1, MTHFR, MTOR, MTPAP, MTR, MTRR, MTTP, MUT, NAA10, NAA35, NAGLU, NAGS, NBAS, NGLY1, NIPA1, NKX2-1, NPC1, NPC2, NPHP1, NT5C2, NUS1, OTC, PAFAH1B1, PAH, PAK3, PALM, PANK2, PCBD1, PCCA, PCCB, PCDH12, PDE10A, PDE2A, PDHA1, PDHB, PDHX, PDP1, PDSS1, PDSS2, PDYN, PEX1, PEX10, PEX11B, PEX12, PEX13, PEX14, PEX16, PEX19, PEX2, PEX26, PEX3, PEX5, PEX6, PHGDH, PHIP, PIGN, PIGT, PLA2G6, PLD3, PLP1, PLXNA2, PMM2, PNKD, PNP, PNPLA6, PNPO, POLG, POLR3A, PPT1, PRKRA, PROSC, PRRT2, PRUNE1, PSAT1, PSPH, PTPN11, PTS, PURA, QDPR, RAB3GAP1, RAB3GAP2, RANBP2, REEP1, REEP2, RHOBTB2, RNASEH2A, RNASEH2B, RNASEH2C, RNASET2, RTN2, SACS, SAMHD1, SATB2, SCN1A, SCN2A, SCN3A, SCN8A, SETD5, SGCE, SGSH, SHH, SIL1, SIX3, SLC16A2, SLC17A5, SLC18A2, SLC19A3, SLC1A4, SLC25A15, SLC25A22, SLC2A1, SLC30A10, SLC33A1, SLC39A14, SLC6A19, SLC6A3, SLC6A5, SLC6A8, SON, SPART, SPAST, SPATA5, SPG11, SPG21, SPG7, SPR, SPTAN1, SPTBN2, SQSTM1, ST3GAL5, STAMBP, STUB1, STXBP1, SUCLA2, SUCLG1, SUOX, SURF1, SYNGAP1, TAF1, TANGO2, TBC1D24, TBCK, TBL1XR1, TCF4, TECPR2, TFG, TGIF1, TGM6, TH, THAP1, TMEM240, TMEM67, TOR1A, TREX1, TRPC3, TSEN54, TTBK2, TTPA, TUBA1A, TUBB2A, TUBB2B, TUBB3, TUBB4A, UBE3A, UCHL1, VAC14, VAMP1, VPS13A, VPS13D, VPS37A, WARS2, WASHC5, WDR45, WDR62, ZBTB18, ZC4H2, ZEB2, ZFR, ZFYVE26, ZIC1, ZIC2, ZIC4* | 424 |
| 9. | Organic acidaemias panel | *ABCD4, ACAD8, ACADSB, ACAT1, ACSF3, ADK, AGK, AHCY, ALDH6A1, AMN, ASPA, ATP5D, AUH, BCKDHA, BCKDHB, BOLA3, BTD, C19ORF70, CBS, CD320, CLPB, CPS1, CUBN, D2HGDH, DBT, DHTKD1, DLD, DNAJC19, ECHS1, ETFA, ETFB, ETFDH, ETHE1, FBP1, FH, FLAD1, FTCD, GCDH, GIF, GLRX5, GLYCTK, GNMT, GSS, HCFC1, HIBCH, HLCS, HMGCL, HSD17B10, HTRA2, IBA57, IDH2, ISCA2, IVD, L2HGDH, LIAS, LIPT1, LIPT2, LMBRD1, MAT1A, MCCC1, MCCC2, MCEE, MLYCD, MMAA, MMAB, MMACHC, MMADHC, MTHFR, MTR, MTRR, MUT, NFU1, OGDH, OPA3, OPLAH, OXCT1, PCCA, PCCB, PCK1, POLG, PPM1K, PRDX1, SERAC1, SLC13A3, SLC13A5, SLC25A1, SLC25A19, SLC25A32, SLC52A1, SLC52A2, SLC52A3, SUCLA2, SUCLG1, SUGCT, TAZ, TCN1, TCN2, THAP11, TIMM50, TMEM70, ZNF143* | 101 |
| 10. | Hereditary spastic paraplegia panel | *ABCD1, ALDH18A1, ALS2, AP4B1, AP4E1, AP4M1, AP4S1, AP5Z1, ARG1, ARL6IP1, ATL1, ATP13A2, B4GALNT1, BSCL2, C12ORF65, CAPN1, CPT1C, CYP27A1, CYP2U1, CYP7B1, DDHD1, DDHD2, ENTPD1, ERLIN1, ERLIN2, FA2H, FARS2, GBA2, GJC2, HACE1, HEXA, HSPD1, KCNA2, KDM5C, KIDINS220, KIF1A, KIF1C, KIF5A, L1CAM, MAG, NIPA1, NKX6-2, NT5C2, PLP1, PNPLA6, RAB3GAP2, REEP1, REEP2, RTN2, SACS, SLC16A2, SPART, SPAST, SPG11, SPG21, SPG7, TECPR2, TFG, UCHL1, VAMP1, WASHC5, ZFYVE26* | 62 |
| 11. | Spinal muscular atrophy panel | *SMN1, SMN2* | 2 |
| 12. | Kabuki panel | *KDM6A, KMT2D* | 2 |
| 13. | Comprehensive myopathy panel | *CTA1, ADSSL1, AMPD1, ANO5, ATP2A1, BAG3, BIN1, CACNA1S, CASQ1, CAV3, CCDC78, CFL2, CLCN1, CNTN1, COL12A1, COL6A1, COL6A2, COL6A3, CPT2, CRYAB, DES, DNAJB6, DNM2, DYSF, FHL1, FKBP14, FLNC, GNE, GYG1, GYS1, HACD1, HNRNPA2B1, ISCU, KBTBD13, KCNJ2, KLHL40, KLHL41, LAMP2, LDB3, LMNA, LMOD3, MAP3K20, MATR3, MEGF10, MICU1, MTM1, MYH2, MYH7, MYL2, MYO18B, MYOT, MYPN, NEB, ORAI1, PYROXD1, RYR1, SCN4A, SELENON, SMPX, SPEG, SQSTM1, STAC3, STIM1, TAZ, TIA1, TK2, TNNT1, TPM2, TPM3, TTN, VCP, VMA21* | 72 |
| 14. | Lysosomal storage disorders panel | *AGA, ARSA, ARSB, ASAH1, ASPA, ATP13A2, CHIT1, CLCN7, CLN2 (TPP1), CLN3, CLN5, CLN6, CLN8, CTNS, CTSA, CTSD, CTSK, FUCA1, GAA, GALC, GALNS, GBA, GLA, GLB1, GM2A, GNE, GNPTAB, GNPTG, GNS, GUSB, HEXA, HEXB, HGSNAT, HYAL1, IDS, IDUA, KCTD7, LAMP2, LIPA, LYST, MAN2B1, MANBA, MCOLN1, MFSD8, MMP14, NAGA, NAGLU, NEU1, NPC1, NPC2, PPT1, PSAP, SCARB2, SGSH, SLC17A5, SMPD1, SUMF1, VPS33A* | 58 |
| 15. | Rett/Angelman like variants | *ADSL, ALDH5A1, ARX, ATRX, CAMK2B, CDKL5, CLTC, CNTNAP2, CTNNB1, DDX3X, DYRK1A, EHMT1, FOLR1, FOXG1, GABBR2, GRIA3, GRIN2A, GRIN2B, HDAC8, IQSEC2, KANSL1, KCNA2, MBD5, MECP2, MEF2C, NGLY1, NRXN1, SATB2, SCN2A, SCN8A, SLC6A1, SLC9A6, SMC1A, STXBP1, SYNGAP1, TBL1XR1, TCF4, UBE3A, WDR45, ZEB2* | 40 |
| 16. | Dystrophinopathies | *DMD* | 1 |
| 17. | NF1 | *NF1* | 1 |
| 18. | RASopathies | *A2ML1, ACTB, ACTG1, BRAF, CBL, HRAS, KAT6B, KRAS, LZTR1, MAP2K1, MAP2K2, MRAS, NF1, NRAS, NSUN2, PPP1CB, PTPN11, RAF1, RASA1, RASA2, RIT1, RRAS, RRAS2, SHOC2, SOS1, SOS2, SPRED1, YWHAZ* | 28 |
| 19. | Holoprosencephaly panel | *FGFR1, GLI2, SHH, SIX3, TGIF1, ZIC2* | 6 |
| 20. | Zellweger spectrum disorders panel | *ACBD5, ACOX1, AMACR, HSD17B4, PEX1, PEX10, PEX11B, PEX12, PEX13, PEX14, PEX16, PEX19, PEX2, PEX26, PEX3, PEX5, PEX6, PEX7* | 18 |
| 21. | Comprehensive muscular dystrophy panel | *ANO5, B3GALNT2, B4GAT1, CAPN3, CAV3, CHKB, COL12A1, COL6A1, COL6A2, COL6A3, DAG1, DES, DMD, DNAJB6, DPM1, DPM2, DPM3, DYSF, EMD, FHL1, FKRP, FKTN, GAA, GMPPB, GOSR2, HNRNPDL, ISPD, ITGA7, LAMA2, LARGE1, LMNA, MYOT, PLEC, PNPLA2, POGLUT1, POMGNT1, POMGNT2, POMK, POMT1, POMT2, RXYLT1, SGCA, SGCB, SGCD, SGCG, SMCHD1, TCAP, TK2, TNPO3, TOR1AIP1, TRAPPC11, TRIM32, TTN* | 53 |
| 22. | Dystonia comprehensive panel | *ACTB, ADCY5, ANO3, ATP1A3, ATP7B, BCAP31, CIZ1, COL6A3, CYP27A1, GCH1, GNAL, GNAO1, HEXA, HPCA, KCNMA1, KCTD17, KMT2B, MECR, PANK2, PLA2G6, PNKD, PRKN, PRKRA, PRRT2, SGCE, SLC2A1, SLC30A10, SLC39A14, SLC6A3, SPR, TH, THAP1, TOR1A, TUBB4A, VAC14, VPS13A, VPS13D, XPR1* | 38 |
| 23. | Congenital muscular dystrophy | *B3GALNT2, B4GAT1, CHKB, COL12A1, COL6A1, COL6A2, COL6A3, DAG1, DMD, DPM1, DPM2, DPM3, FKRP, FKTN, GMPPB, GOSR2, ISPD, ITGA7, LAMA2, LARGE1, LMNA, POMGNT1, POMGNT2, POMK, POMT1, POMT2, RXYLT1, TCAP, TK2* | 29 |
| 24. | Elevated Glycine panel | *AMT, BOLA3, GLDC, GLRX5, IBA57, ISCA2, LIAS, LIPT1, LIPT2, NFU1, PNPO, SLC6A9* | 12 |
