# Supplementary material for: Utility of next generation sequencing in paediatric neurological disorders: experience from South Africa
Source: Eur J Hum Genet. 2024 May 3;32(10):1314–8. doi: 10.1038/s41431-024-01582-2 (PMC11499987; doi:10.1038/s41431-024-01582-2)
Supplement: Supplementary file 2 — Supplementary Table 2 [file 41431_2024_1582_MOESM2_ESM.docx]

**Supplementary Table 2: Number of pathogenic variants identified per gene/gene panel**

|  | **Panel** | **Gene** | **Number of patients with pathogenic variants** |
| --- | --- | --- | --- |
| 1. | Epilepsy Panel | *SCN1A* | 7 |
|  |  | *COG5* | 1** |
|  |  | *DYRK1A* | 1 |
|  |  | *GABRB3* | 1 |
|  |  | *UBE3A* | 1* |
|  |  | *STXBP1* | 1 |
|  |  | *KANSL1* | 1 |
|  |  | *SCN2A* | 1 |
|  |  | *TPP1* | 1 |
|  |  | *CDKL5* | 3 |
|  |  | *KCNQ2* | 3 |
|  |  | *KCNMA1* | 1 |
|  |  | *GNAO1* | 1 |
|  |  | *SLC6A5* | 1 |
| 2. | Comprehensive neuromuscular panel | *RYR1* | 2 |
|  |  | *SMN1* | 1 |
|  |  | *TTN* | 1** |
|  |  | *GBE1* | 1 |
|  |  | *CAPN3* | 1* |
|  |  | *STAC3* | 1 |
|  |  | *DMD* | 1 |
| 3. | Early infantile epileptic encephalopathy panel | *CDKL5* | 1 |
| 4. | Hereditary sensory and autonomic neuropathy panel | *SCN9A* | 2 |
| 5. | Leuko-dystrophy and -encephalopathy panel | *ACADS* | 1** |
|  |  | *UGT1A1* | 2* |
|  |  | *ABCD1* | 1 |
|  |  | *PLP1* | 1 |
|  |  | *ARSA* | 1 |
| 6. | Metachromatic and general leukoencephalopathy panel | *ARSA* | 1 |
| 7. | Cerebral palsy spectrum disorders panel | *SCN2A* | 1 |
|  |  | *ADAR* | 1+1* |
|  |  | *BTD* | 1* |
|  |  | *SLC16A2* | 1 |
|  |  | *KCNA2* | 1 |
|  |  | *QDPR* | 1 |
|  |  | *ATM* | 1* |
|  |  | *NPHP1* | 1* |
| 8. | Organic acidaemias panel | *PCCA* | 1 |
| 9. | Spinal muscular atrophy panel | *SMN1, SMN2* | 1 |
| 10. | Comprehensive myopathy panel | *STAC3* | 1 |
|  |  | *RYR1* | 1 |
| 11. | Rett/Angelman like variants | *IQSEC2* | 1 |
|  |  | *GABBR2* | 1 |
| 12. | Dystrophinopathies | *DMD* | 1 |
| 13. | NF1 | *NF1* | 1 |
| 14. | RASopathies | *NF1* | 1 |
| 15. | Zellweger spectrum disorders panel | *HSD17B4* | 1** |
| 16. | Comprehensive muscular dystrophy panel | *DMD* | 2 |
|  |  | *COL6A1* | 1 |
| 17. | Dystonia comprehensive panel | *KMT2B* | 1 |

*Pathogenic variants not considered to contribute to the phenotype

** Pathogenic variant in a gene known to cause autosomal-recessively inherited disease
