# Supplementary material for: Utility of next generation sequencing in paediatric neurological disorders: experience from South Africa
Source: Eur J Hum Genet. 2024 May 3;32(10):1314–8. doi: 10.1038/s41431-024-01582-2 (PMC11499987; doi:10.1038/s41431-024-01582-2)
Supplement: Supplementary file 3 — Supplementary Table 3. [file 41431_2024_1582_MOESM3_ESM.docx]

**Supplementary Table 3. Diagnostic yield per panel and neurological disease group.**

| PANELS | Number of panels requested | Gene | Number of pathogenic variants identified | Number with phenotypic correlation |
| --- | --- | --- | --- | --- |
| **DISORDER GROUPS** |  |  |  |  |
| **Neuromuscular**  **disorders** |  |  |  |  |
| Comprehensive neuromuscular panel | 14 | *RYR1* | 2 | 2 |
|  |  | *SMN1* | 1 | 1 |
|  |  | *TTN* | 1 | 0 |
|  |  | *CAPN3* | 1 | 0 |
|  |  | *GBE1* | 1 | 1 |
|  |  | *STAC3* | 1 | 1 |
|  |  | *DMD* | 1 | 1 |
|  |  |  | **8(57%)** | **6(43%)** |
| Comprehensive neuropathies panel | 1 |  | 0 | 0 |
| Hereditary sensory and autonomic neuropathy panel | 2 | *SCN9A* | 2 | 2 |
|  |  |  | **2(100%)** | **2(100%)** |
| Spinal muscular atrophy panel | 1 | *SMN1, SMNcopies2* | 1 | 1 |
|  |  |  | **1(100%)** | **1(100%)** |
| Comprehensive myopathy panel | 2 | *STAC3* | 1 | 1 |
|  |  | *RYR1* | 1 | 1 |
|  |  |  | **2(100%)** | **2(100%)** |
| Dystrophinopathies | 1 | *DMD* | 1 | 1 |
|  |  |  | **1(100%)** | **1(100%)** |
| Comprehensive muscular dystrophy panel | 3 | *DMD* | 2 | 2 |
|  |  | *COL6A1* | 1 | 1 |
|  |  |  | **3(100%)** | **3(100%)** |
| Congenital muscular dystrophy | 1 |  | 0 | 0 |
| **TOTAL PER GROUP** | **25** |  | **17(68%)** | **15(52%)** |
|  |  |  |  |  |
| **Epilepsies** |  |  |  |  |
| Epilepsy panel | 54 | *SCN1A* | 6 | 6 |
|  |  | *COG5* | 1 | 0 |
|  |  | *DYRK1A* | 1 | 1 |
|  |  | *GABRB3* | 1 | 1 |
|  |  | *UBE3A* | 1 | 0 |
|  |  | *STXBP1* | 1 | 1 |
|  |  | *KCNMA1* | 1 | 1 |
|  |  | *KANSL1* | 1 | 1 |
|  |  | *SCN2A* | 1 | 1 |
|  |  | *TPP1* | 1 | 1 |
|  |  | *CDKL5* | 3 | 3 |
|  |  | *KCNQ2* | 3 | 3 |
|  |  | *GNAO1* | 1 | 1 |
|  |  | *SLC6A5* | 1 | 1 |
|  |  | *NPRL3* | 1 | 1 |
|  |  | *PCDH19* | 1 | 1 |
|  |  |  | **25 (46%)** | **23 (43%)** |
| Early infantile epileptic encephalopathy panel | 9 | *CDKL5* | 1 | 1 |
|  |  | *SCN1A* | 1 | 1 |
|  |  | *SMC1A* | 1 | 1 |
|  |  |  | **3(33%)** | **3(33%)** |
| **TOTAL PER GROUP** | **63** |  | **28 (44%)** | **26(41%)** |
| **Movement disorders** |  |  |  |  |
| Dystonia comprehensive panel | 1 | *KMT2B* | 1 | 1 |
| **TOTAL PER GROUP** | **1** |  | **1(100%)** | **1(100%)** |
| **Heredodegenerative disorders** |  |  |  |  |
| Metachromatic and general leukoencephalopathy panel | 2 | *ARSA* | 1 | 1 |
|  |  |  | **1(50%)** | **1(50%)** |
| Leukodystrophy and leukoencephalopathy panel | 6 | *ACAD5* | 1 | 0 |
|  |  | *UGT1A1* | 1 | 0 |
|  |  | *ABCD1* | 1 | 1 |
|  |  | *UGT1A1* | 1 | 0 |
|  |  | *PLP1* | 1 | 1 |
|  |  | *ARSA* | 1 | 1 |
|  |  |  | **6(100%)** | **3(50%)** |
| Hereditary spastic paraplegia panel | 1 |  | 0 | 0 |
| **TOTAL PER GROUP** | **9** |  | **7(77%)** | **4(44%)** |
| **Neurocutaneous disorders** |  |  |  |  |
| Neurofibromatosis 1 | 1 | *NF1* | 1 | 1 |
| **TOTAL PER GROUP** | **1** |  | **1(100%)** | **1(100%)** |
| **Metabolic disorders** |  |  |  |  |
| Lysosomal storage disorders panel | 1 |  | 0 | 0 |
| Organic acidaemias panel | 1 | *PCCA* | 1 | 1 |
|  |  |  | **1(100%)** | **1(100%)** |
| Zellweger spectrum disorders panel | 1 | *HSD17B4* | 1 | 0 |
|  |  |  | **1(100%)** | **0** |
| Glycine encephalopathy | 1 |  | 0 | 0 |
| **TOTAL PER GROUP** | **4** |  | **2(50%)** | **1(25%)** |
| **Cerebral palsy spectrum disorders** |  |  |  |  |
| Cerebral palsy spectrum disorders panel | 16 | *SCN2A* | 1 | 1 |
|  |  | *ADAR* | 2 | 1 |
|  |  | *SLC16A2* | 1 | 1 |
|  |  | *BTD* | 2 | 0 |
|  |  | *KCNA2* | 1 | 1 |
|  |  | *QDPR* | 1 | 1 |
|  |  | *ATM* | 1 | 0 |
|  |  | *NPHP1* | 1 | 0 |
| **TOTAL PER GROUP** | **16** |  | **10(63%)** | **5 (31%)** |
| **Neuro-developmental disorders** |  |  |  |  |
| Rett/Angelman like variants | 3 | *IQSEC2* | 1 | 1 |
|  |  | *GABBR2* | 1 | 1 |
|  |  |  | **2(66%)** | **2(66%)** |
| Kabuki panel | 1 |  | 0 | 0 |
| RASopathies | 1 | *NF1* | 1 | 1 |
|  |  |  | **1(100%)** | **1(100%)** |
| **TOTAL PER GROUP** | **5** |  | **3(60%)** | **3(60%)** |
| **Developmental brain Malformations** |  |  |  |  |
| Holoprosencephaly panel | 3 |  | 0 | 0 |
| **TOTAL PER GROUP** | **3** |  | **0** | **0** |
| **TOTAL NUMBERS** | **127** |  | **68(54%)** | **56(44%)** |
|  |  |  |  |  |
|  |  |  |  |  |
