# Supplementary material for: Utility of next generation sequencing in paediatric neurological disorders: experience from South Africa
Source: Eur J Hum Genet. 2024 May 3;32(10):1314–8. doi: 10.1038/s41431-024-01582-2 (PMC11499987; doi:10.1038/s41431-024-01582-2)
Supplement: Supplementary file 4 — Supplementary Table 4 [file 41431_2024_1582_MOESM4_ESM.docx]

| Question | YES | NO | Some/Little | Unknown | Not applicable |
| --- | --- | --- | --- | --- | --- |
| Did the result help explain the reason for your child illness? |  |  |  |  |  |
| Were any changes made to the treatment/ medication after the diagnosis? |  |  |  |  |  |
| Did the result help you to take better care for your child? |  |  |  |  |  |
| Did the results help you plan for future pregnancies? |  |  |  |  |  |
| Should prenatal testing become available, would make use of it in next pregnancies? |  |  |  |  |  |
| Did knowing the result bring you and your family any closure? |  |  |  |  |  |

**Supplementary Table 4: Care-giver questionnaire**
