# Supplementary material for: Utility of next generation sequencing in paediatric neurological disorders: experience from South Africa
Source: Eur J Hum Genet. 2024 May 3;32(10):1314–8. doi: 10.1038/s41431-024-01582-2 (PMC11499987; doi:10.1038/s41431-024-01582-2)
Supplement: Supplementary file 5 — Supplementary Table 5 [file 41431_2024_1582_MOESM5_ESM.docx]

**Supplementary Table 5: Patients diagnosed- Pathogenic variants identified with information on precision therapy (4)​**

| Patient | Phenotypic features | Gene | OMIM | Inheritance/ zygosity | ACMG Classification | ACMG 2015 Criteria | | Precision Therapy​(8,15)​ | Therapy implemented or adjusted |
| --- | --- | --- | --- | --- | --- | --- | --- | --- | --- |
| 69  (M) | Subacute onset of dense left hemiplegia. MRI features suggestive of adrenoleukodystrophy | *ABCD1*  NM_000033.4: c.1415_1416del (p.Gln472Argfs*83) | #300100 | X-linked /  hemizygous | Pathogenic | PVS1, PP5, PM2 | | Hemato-poietic stem cell transplant | Supportive therapy |
| 68(M) | Childhood-onset dystonia, cyclical transaminitis and freckling of the skin. Bilateral striatal necrosis on MRI brain | *ADAR*  NM_001111.4: c.2128_2131dup (p.Asn711Thrfs*34)   VCV001452076.2  *ADAR*  NM_001111.4: c.577C>G (p.Pro193Ala)  VCV000126395.65 | #146920 | AR/  Compound heterozygous | Pathogenic  Also identified in mother and brother who have AD-related disease.    VUS – in trans with the Pathogenic variant, identified in healthy father | PVS1, PM2, PP3,  PP4          PS3,  PS4, PM2, PM3 | | None | Biotin,  Baclofen |
| 60(M) | Developmental delay and epilepsy then neurodevelopmental regression and progressive vision loss, MRI leukoencephalopathy | *ARSA*  NM_000487.5: c.905G>A (p.Cys302Tyr)  VCV000963270.8 | #607574 | AR/  homozygous | Pathogenic | PP3,  PP5, PM1, PM5, PM2 | | None | Baclofen and Sodium Valproate |
| 92(M) | 4-and-a-half-year-old with 2 episodes of ataxia, resolved, developed dystonia and spastic diplegia in 2021, now with regressing milestones. | *ARSA*  NM_000487.5: c.1150G>A (p.Glu384Lys) and   VCV000003084.26  *ARSA*  NM_000487.5:  c.905G>A (p.Cys302Tyr  VCV000963270.8 | #607574 | AR/  Compound  heterozygous | Pathogenic            Pathogenic | PP5, PM3, PM2, PP3        PP3,  PP5, PM1, PM5, PM2 | | None | Levo-dopa: no effect.  Low dose Carbamazepine |
| 100  (M) | Infantile spasms. EEG: Hypsarrhythmia  Severe developmental delay, acquired microcephaly, cortical blindness. | *CDKL5*  NM_001323289.2: c.175C>T (p.Arg59*)  VCV000143783.14 | #300672 | X-linked  hemizygous | Pathogenic | PVS1, PM2, PP3,  PP4 | | Ganaloxone | ACTH, Phenobarbitone, Clonazepam. Then Sodium Valproate and Lamotrigine when older.  Ganalaxone not available |
| 77  (F) | Seizures from 6 weeks of age. EEG: Hypsarrhythmia  Acquired microcephaly, Hand-wringing | *CDKL5*  NM_001323289.2: c.1927C>T (p.Gln643*)  VCV001070908.2 | #300672 | X-linked  heterozygous | Pathogenic | PVS1, PM2, PP3,  PP4 | | Ganaloxone | Prednisone. Vigabatrin, Pyridoxine, Phenobarb  Ganalaxone not available |
| DEE1(F) | Microcephaly  Central hypotonia  not fixing and following  Mouthing and hand wringing.  Severe developmental delay.  Myoclonic, tonic and GTC seizures | *CDKL5*  NM_001323289.2: c.2360_2363del (p.Lys787Argfs*15)   VCV000156651.7 | #300672 | X-linked/  heterozygous | Pathogenic | PVS1, PM2, PP3,  PP4 | | Ganaloxone | Sodium valproate/clobazam /levetiracetam  Now on ketogenic diet  Ganalaxone not available |
| DEE2(F) | Acquired microcephaly  Central and peripheral hypotonia  Not fixing and following  Severe developmental delay.Acquired microcephaly.  Central and peripheral hypotonia.  Not fixing and following  Severe developmental delay | *CDKL5*  NM_001323289.2: c.1927C>T (p.Gln643*)  VCV001070908.2 | #300672 | X-linked/  heterozygous | Pathogenic | PVS1, PM2, PP3,  PP4 | | Ganaloxone | Sodium valproate/  clobazam/  lamotrigine  Ganaloxone not avialable |
| 34(M) | Progressive lower limb weakness and positive Gower sign, no pseudohypertrophy of calves, CK600 | *COL6A1*  NM_001848.3: c.868G>A (p.Gly290Arg)  VCV000093894.60 | #120220 | AD/  heterozygous | Pathogenic | PS3,  PS4, PM2 | | Supportive therapy | Supportive therapy |
| 33(M) | Progressive weakness and positive Gower sign, CK >4000 | *DMD*  NM_004006.2: c.9204_9207del (p.Asn3068Lysfs*20)  VCV000217217.21 | #300376 | X-linked/  hemizygous | Pathogenic | PVS1, PP5, PM2 | | Steroids  Enalapril  Gene therapies | Prednisone (Deflazacort not available)  Enalapril, gene therapies not available |
| 38(M) | Progressive proximal weakness and positive Gower sign, high CK | *DMD*  NM_004006.2: Deletion (Exons 63-79)  VCV002424920.4 | #300376 | X-linked  hemizygous | Pathogenic | PVS1,  PS3, PS4, PM2, PM4, PP3 | | Steroids  Enalapril  Gene therapies | Prednisone (Deflazacort not available)  Enalapril, Gene therapies not available |
| 65(M) | Normal development and then progressive lower limb weakness with positive Gower sign | *DMD*  NM_004006.2: c.2695G>T (p.Glu899*)  VCV001073002.8 | #300376 | X-linked  hemizygous | Pathogenic | PVS1, PP5, PM2 | | Steroids  Enalapril  Gene therapies | Prednisone (Deflazacort not available)  Enalapril, Gene therapies not available |
| 110(M) | Previously normal development, now proximal weakness and clumsiness when walking. Hypertrophy of the claves. | *DMD*  NM_004006.2: c.4996C>T (p.Arg1666*)  VCV000094646.20 | #300376 | X-linked  hemizygous | Pathogenic | PVS1, PP5, PM2 | | Steroids  Enalapril  Gene therapies | Prednisone (Deflazacort not available)  Enalapril  Gene therapies not available |
| 9  (F) | Epilepsy, Moderate developmental delay and microcephaly | *DYRK1A*  NM_001347721.2:  c.957C>A (p.Tyr319*)  VCV001918259.2 | #614140 | AD/  heterozygous | Pathogenic | PVS1, PM2, PP3, PP4 | | None | Lamotrigine |
| 89(F) | Developmental delay, minimal speech, autistic-like features, microcephaly, unusual hand movements. No significant family history | *GABBR2*  NM_005458.8: c.1699G>A (p.Ala567Thr)  VCV 000446211.33 | #607340 | AD/  heterozygous | Pathogenic | PP5, PM5, PP3, PM2 | | None | Supportive therapy |
| 14  (M) | Seizures from 10month of age. Multiple seizure types, refractory to multiple ASM,  Microcephalic, happy demeanour and global developmental delay, non-verbal.  (see supplementary table 5) | *GABRB3*  NM_000814.5:  Deletion  (entire coding sequence deleted)     VCV002685503.1 | #617113 | AD/heterozygous | Pathogenic | PVS1, PM2, PP3, PP4 | LoF: GABAergic enhancers eg Phenobarbital and Vigabatrin GoF: avoid GABAergic enhancers | | Sodium Valproate and Clobazam and Risperidone |
| 70(M) | Weak, low Apgar score, contractures, ventilated for 15 days in NICU. | *GBE1*  NM_000158.4: c.1854del (p.Ala619Glnfs*32)  VCV002065260.1 | #607839 | AR/ homozygous | Pathogenic | PVS1, PS4, PM2 | None | | Supportive therapy |
| DEE3(F) | Fixing and following.  No chorea or abnormal movements noticed.  Moderate developmental delay. Multiple left focal seizures, followed by frequent generalised tonic clonic seizures. | *GNAO1*  NM_020988.3: c.871T>A  (p.Tyr291Asn)  VCV000421826.6 | #139311 | AD/  heterozygous | Pathogenic | PS1, PS3, PS4, PM1, PM2 | None | | Phenobarbitone/ pyridoxine |
| DEE5(F) | Autistic spectrum, cognitive regression after the 1st seizure  Nocturnal GTC (5 – 10 min) | *IQSEC2*  NM_001111125.2: c.2026del (p.Ala676Leufs*46)  VCV000850650.3 | #300522 | X-linked /  hemizygous | Pathogenic | PVS1, PS4, PM2 | None | | Sodium valproate/ lamotrigine/clobazam  Now on Phenobarbitone and lamotrigine |
| DEE6(F) | Autistic spectrum  Severe developmental delay  epileptic spasms, myoclonic, atonic, focal with secondary generalization. | *KANSL1*  NM_001379198.1:  c.2542-1G>A  VCV001333700.5 | #612452 | AD/ heterozygous | Likely Pathogenic | PVS1, PS4, PM2 | None | | Sodium valproate/  clobazam /levetiracetam/vigabatrin /pyridoxine |
| 93(M) | Seizures, frontal cortical dysplasia, developmental delay, and progressive spastic paraplegia | *KCNA2*  NM_001204269.2: c.881G>C (p.Arg294Pro)  VCV002109320.2 | #176262 | AD/  heterozygous | Likely Pathogenic | PM1, PM2, PM5 | GoF: 4-Aminopyridine,  Sodium Channel Blockers. No specific therapy for LoF. | | Carbamazepine |
| DEE8(F) | Left paralytic strabismus  Central hypotonia  Truncal instability, broad based gait, complex febrile seizures (right focal), then status epilepticus | *KCNMA1*  NM_002247.4: c.1284del (p.Leu429Cysfs*53)  VCV001414943.1 | #600150 | AD/  heterozygous | Pathogenic | PVS1, PS4, PM2 | None | | Sodium valproate/ lamotrigine |
| 104  (M) | Early onset neonatal seizures. Refractory.  Floppy strong with Developmental delay. Seizures resolved at 3y age.  Dystonia | *KCNQ2*  NM_172107.4: c.740C>T (p.Ser247Leu)  VCV000205878.10 | #602235 | AD/  heterozygous | Pathogenic | PP5, PM1, PM5, PP3, PM2 | LoF: Sodium Channel Blockers | | Phenobarb, Clonazepam, Pyridoxine. Changed to Epilim at 6months of age. Now Baclofen |
| DEE9(F) | Normocephalic  Central hypotonia  Severe developmental delay  Eyelid twitching  Bilateral tonic seizures | *KCNQ2*  NM_172107.4: c.1688A>G (p.Asp563Gly)  VCV000836158.6 | #602235 | AD/  heterozygous | Likely Pathogenic | PM1, PM2, PM5 | LoF: Sodium Channel Blockers | | Sodium valproate/ lamotrigine  Now on carbamazepine |
| DEE10  (M) | Normal exam  Mild – moderate delay  Focal seizures | *KCNQ2*  NM_172107.4: Deletion  (Exons 8-15) | #602235 | AD/  heterozygous | Pathogenic | PVS1, PS4, PM2 | LoF: sodium Channel Blockers | | Phenobarbitone |
| 97(F) | Previously normal development presenting with progressive dystonia | *KMT2B*  NM_014727.3: c.2200_2201del (p.Gln734Glyfs*111) VCV002132480.2 | #617284 | AD/  heterozygous | Pathogenic | PVS1, PM2, PM4 | Deep Brain stimulation | | Baclofen and Diazepam |
| 71(M) | Multiple CALs, neurofibromas with intellectual disability | *NF1*  NM_000267.3: c.4537C>T (p.Arg1513*)  VCV000220152.69 | #613113 | AD/  heterozygous | Pathogenic | PVS1, PS3, PS4 | None | | No Therapy |
| 72  (M) | Pulmonary stenosis Multiple CALs | *NF1*  NM_000267.3: Deletion (Exons 16-17) | #613113 | AD/  heterozygous | Pathogenic | PVS1, PS3, PS4 | None | | No Therapy |
| 122  (F) | Abrupt onset Childhood onset focal motor seizures with generalisation, up to 30 times per day. EEG confirmed semiology. Normal MRI. Normal development | *NPRL3*  NM_001243249.1: c.119-1G>C (Splice acceptor)  VCV001465304.7 | #600928 | AD  heterozygous | Likely Pathogenic | PVS1, PM2, | MTOR inhibitors,  Resective surgery | | Immune  Modulation, Levetiracetam. |
| 79(F) | Previously well infant presented with infantile encephalopathy and raised ammonia levels | *PCCA*  NM_000281.3: c.1288C>T (p.Arg430*)  VCV000851298.9  and  NM_000281.3: c.1593_1595del (p.Leu532del)  VCV000554477.13 | #232000 | AR/Compound  heterozygous | Pathogenic          Likely Pathogenic | PVS1, PM2, PS4      PVS1, PM2, PM3 | Acute management protocol,  Carnitine | | Acute management protocol,  Carnitine |
| DEE11 (M) | Global developmental delay  Severe intellectual disability  Motor restlessness  Presented with right focal status, 3 months later GTC seizures.  Now clusters of seizures once per month, tonic with eye deviation | *PCDH19*  NM_001105243.2: c.1031C>G(p.Pro344Arg)  VCV001507139.8 | #603625 | X-linked  hemizygous | Likely Pathogenic | PM1, PM2, PM5 | None | | Sodium valproate/ lamotrigine    Now Clobazam |
| 73(M) | Developmental delay, intellectual disability. Hypomyelination on MRI | *PLP1*  NM_000533.4: Whole gene duplication | #300401 | X-linked  hemizygous | Pathogenic | PS3, PS4, PM2, PM4, PP3, PP4 | None | | Supportive therapy |
| 94(F) | CP-like presentation but normal Birth history and two other siblings with similar presentation | *QDPR*  NM_000320.3: c.196C>T (p.Gln66*)  VCV002128493.2 | #612676 | AR/  homozygous | Pathogenic | PVS1, PS3, PM2, PP3, PP4 | Tetrahydrobiopterin | | Patient demised |
| 21(M) | Floppy weak infant | *RYR1*  NM_000540.2: c.10348-6C>G (Intronic)   VCV000132994.40  *RYR1*  NM_000540.2: c.9001-12A>G (Intronic)   VCV001472955.3 | #180901 | AR/  Compound heterozygous | Pathogenic  (inherited from mother)          VUS (not inherited from mother, father unavailable) | PP5, PS4,  PM2, PP4            PM2, PM3, PP4 | Avoid Succinylcholine and certain anaesthetic inhalants | | Avoid Succinylcholine and certain anaesthetic inhalants |
| 62(F) | Floppy weak infant with facial diplegia | *RYR1*  NM_000540.2: c.10348-6C>G (Intronic)   VCV000132994.40  *RYR1*  NM_000540.2: c.223C>T (p.Arg75*)  VCV000544401.6 | #180901 | AR/Compound  heterozygous | Pathogenic              Pathogenic | PP5, PS4,  PM2, PP4        PVS1, PS4, PM2, PM3, PP4 | Avoid Succinylcholine and certain anaesthetic inhalants | | Avoid Succinylcholine and certain anaesthetic inhalants |
| 67(F) | Floppy weak infant, dysmorphic features, facial diplegia, micrognathia, low-set ears, prolonged oxygen support needed, feeding difficulties, multiple admissions with respiratory distress | *RYR1*  NM_000540.2: c.10348-6C>G (Intronic)   VCV000132994.40      *RYR1*  NM_000540.2: c.6797-1G>A (Splice acceptor)  VCV001067810.7 | #180901 | AR/Compound  heterozygous | Pathogenic              Pathogenic | PP5, PS4,  PM2, PP4        PVS1, PM2, PM3, PP4 | Avoid Succinylcholine and certain anaesthetic inhalants | | Avoid Succinylcholine and certain anaesthetic inhalants |
| 91(M) | Infantile onset febrile seizures, also focal seizures tonic clinic and generalised tonic seizures. Initial EEG normal | *SCN1A*  NM_001165963.1: c.3879+1G>A (Splice donor)  VCV002203184.2 | #182389 | AD/  heterozygous | Pathogenic | PVS1, PS4, PM2, PM6 | GoF: Avoid Sodium Channel Blockers  LoF: Stiripentol,  fenfluramine, cannabidiol (Valproate Clobazam) | | Sodium valproate, levetiracetam,  Clobazam  Fenfluramine Cannabidiol not available |
| 111  (M) | Early onset seizures (3months), developmental delay | *SCN1A*  NM_001165963.1: c.2836C>T (p.Arg946Cys)  VCV000068604.36 | #182389 | AD/  heterozygous | Pathogenic | PP5, PM1, PM5, PP3, PM2 | GoF: Avoid Sodium Channel Blockers  LoF: Stiripentol,  fenfluramine, cannabidiol (Valproate Clobazam | | Phenobarbitone then Sodium Valproate and Levetiracetam  Fenfluramine Cannabidiol not available |
| DEE12 (M) | Focal and GTC with fever, subsequently unprovoked, multiple seizures with status epilepticus. Development delay – severe | *SCN1A*  NM_001165963.1: c.3733C>T (p.Arg1245*)  VCV000167639.36 | #182389 | AD/  heterozygous | Pathogenic | PVS1, PS4, PM2 | GoF: Avoid Sodium Channel Blockers  LoF: Stiripentol, Fenfluramine, cannabidiol (Valproate Clobazam) | | Sodium valproate/ clobazam/ levetiracetam. Fenfluramine Cannabidiol not availabe    Lamotrigine(removed) |
| DEE13  (M) | Initially normal development, Left focal seizures, initially with fever, subsequently multiple focal status, then GTC. Subsequently developmental delay, with autistic features, marked speech delay. Father had febrile seizures | *SCN1A*  NM_001165963.1: c.4547C>A (p.Ser1516*)  VCV000206837.16 | #182389 | AD/  heterozygous | Pathogenic | PVS1, PS4, PM2 | GoF: Avoid Sodium Channel Blockers  LoF: Stiripentol,  Fenfluramine, cannabidiol (Valproate Clobazam) | | Sodium valproate /lamotrigine/levetiracetam,  worsened on carbamazepine.  Fenfluramine Cannabidiol not available |
| DEE14  (M) | Normocephalic, hyperactive, no focal neurology  Developmental delay – moderate. Initially GTC unprovoked, now left focal, seizures worsened on lamotrigine | *SCN1A*  NM_001165963.1: c.4168G>A, p.Val1390Met  VCV000068537.15 | #182389 | AD/ heterozygous | Pathogenic | PM1, PM2, PP3, PM5 | GoF: Avoid Sodium Channel Blockers  LoF: Stiripentol  Fenfluramine  cannabidiol (Valproate Clobazam) | | Sodium valproate/ levetiracetam Fenfluramine Cannabidiol not available |
| DEE15  (M) | Normocephalic, no focal neurology, motor restlessness.  Developmental delay – severe.  First seizure with fever, subsequent multiple seizures, GTC, left focal, myoclonic, 8/day. Now 2-3 /week | *SCN1A*  NM_001165963.1: c.3438_3442del(p.Asn1146lysfs*3)  VCV001430555.4 | #182389 | AD/  heterozygous | Pathogenic | PVS1, PS4, PM2 | GoF: Avoid Sodium Channel Blockers  LoF: Stiripentol  Fenfluramine, cannabidiol (Valproate Clobazam) | | Sodium valproate/clobazam /levetiracetam.  Fenfluramine Cannabidiol not available |
| DEE16  (M) | Microcephalic (-3 Z score), no life skills, vision and hearing intact.  Developmental delay – severe.  initially febrile seizures, then focal seizures, generalised seizures, myoclonic jerks and atonic seizures | *SCN1A* NM_001165963.1: c.4128T>G (p.Cys1376Trp)    VCV000946512.2 | #182389 | AD/  heterozygous | Pathogenic | PP5, PM5, PP3, PM1, PM2 | GoF: Avoid Sodium Channel Blockers  LoF: Stiripentol  Fenfluramine, cannabidiol (Valproate Clobazam) | | vigabatrin/clobazam/sodium valproate/levetiracetam. Now seizure free.  Fenfluramine Cannabidiol not available |
| 10(F) | CP-like with central hypotonia and upper motor neuron signs in all limbs. Severe global developmental delay and visual impairment | *SCN2A*  NM_021007.2: c.788C>T (p.Ala263Val)  VCV000029888.63 | #182390 | AD/  heterozygous | Pathogenic | PS3, PS4, PM2, PM6 | GoF Sodium Channel Blockers | | Supportive therapy |
| DEE18  (F) | Microcephaly  Autistic spectrum, regression after epileptic spasms  Severe developmental delay | *SCN2A*  NM_021007.2: c.3631G>A (p.Glu1211Lys)  VCV000029886.22 | #182390 | AD/  heterozygous | Pathogenic | PS3, PS4, PM2, PM6 | GoF Sodium Channel Blockers | | Vigabatrin/ prednisone/ followed by sodium valproate. Now weaned |
| 27(F) | Intellectual disability and insensitivity to pain/Twins | *SCN9A*  NM_002977.3: c.3891+1G>C (Splice donor)  VCV001496453.3 | #603415 | AR/  homozygous | Likely Pathogenic | PVS1, PM2 | None | | Risperidone |
| 28(F) | Intellectual disability and insensitivity to pain/Twins | *SCN9A*  NM_002977.3: c.3891+1G>C (Splice donor)  VCV001496453.3 | #603415 | AR/  homozygous | Likely Pathogenic | PVS1, PM2 | None | | Methylphenidate |
| 88  (M) | Central hypotonia with peripherally increased tone and reflexes. Global developmental delay. MRI demyelination | *SLC16A2*  NM_006517.4: c.407dup (p.Asn136Lysfs*31)  VCV000694739.8 | #300095 | X-Linked  hemizygous | Pathogenic | PVS1, PM2, PP3, PP4 | Avoid Gaba potentiating agents | | Supportive therapy |
| 123(F) | Baby with exaggerated startle and then tonic posturing | *SLC6A5*  NM_004211.3: Deletion (Exon 14)      *SLC6A5*  NM_004211.3: c.1969+4A>T  VCV000643116.4 | #61618 | AR/  Compound heterozygous | Pathogenic  (inherited from mother)      VUS  (in trans – not inherited from mother, father not available) | PVS1, PS4          PS4, PM2, PM3, PP3, PP4 | Clonazepam, carbamazepine, clobazam, phenytoin, diazepam, valproate, 5-hydroxytryptophan, piracetam, and phenobarbital. | | Clonazepam |
| DEE  19  (F) | Mild ataxia worse on the right  Autistic spectrum  Severe developmental delay  GTC, Left focal – secondary generalised. | *SMC1A*  NM_006306.4: c.2314-7_2314-3del (Intronic)  VCV001068223.8 | #300040 | AD/  heterozygous | Likely Pathogenic | PS4, PM2, PP3 | Multiple anti-seizure medications and ketogenic diet | | Sodium valproate/ lamotrigine/ phenytoin |
| 31  (M) | Neonatal onset severe weakness. Requiring respiratory support and NGT feeding | *SMN1*  NM_000344.3:Deletion (Entire coding sequence)  SMN2 copy no. = 2 | #600354 | AR/  Homozygous | Pathogenic | PVS1, PS3, PS4, | Spinraza | | Supportive therapy, Spinraza-Not available |
| 113  (M) | Floppy weak infant, no dysmorphic feature | *SMN1*  NM_000344.3: Deletion (Entire coding sequence)  SMN2 copy no. = 2 | #600354 | AR/  homozygous | Pathogenic | PVS1, PS3, PS4, | Spinraza | | Supportive therapy, Spinraza-Not available |
| 58(F) | Mild hypotonia, cleft palate, wide spaced nipples and intermittent stridor. | *STAC3*  NM_145064.2: c.851G>C (p.Trp284Ser)  VCV000088744.48 | #255995 | AR/  homozygous | Pathogenic | PS3, PS4, PM2 | None | | Supportive Therapy |
| 107  (F) | Floppy weak infant | *STAC3*  NM_145064.2:  c.851G>C (p.Trp284Ser)  VCV000088744.48 | #255995 | AR/  homozygous | Pathogenic | PS3, PS4, PM2 | None | | Supportive therapy |
| DEE  20  (F) | Acquired microcephaly, hyperactive, no focal neurology.  Initially had febrile seizures. Subsequent unprovoked generalized tonic seizures lateral deviation of eye, mouth deviation. mainly nocturnal. | *STXBP1*  NM_001374314.1:  c.1652G>A (p.Arg551His)  VCV000566474.12 | #602926 | AD/  heterozygous | Pathogenic | PS3, PS4, PM2, PP3, PP4 | Levetiracetam may have superior effect | | Sodium valproate/ lamotrigine/clobazam |
| 46  (M) | 5-year-old male with onset of seizures at age 4, followed by progressive developmental regression, progressive visual loss with optic atrophy, ataxic/myoclonic movements and microcephaly. MRI brain: cerebellar atrophy with mild supratentorial atrophy. Metabolic work up normal. | *TPP1*  NM_000391.3:  c.1424C>T (p.Ser475Leu)  VCV000068741.16 | #607998 | AR/ homozygous | Pathogenic | PS1, PS3, PM2, PP3, PP5 | Cerliponase alfa | | Clobazam, Baclofen  Cerliponase alfa not available |

”DEE” patients included from previous study ​(4)​

AR- autosomal recessive, AD- Autosomal dominant, CALs- Café au lait spots, CK- Creatine kinase, CP- Cerebral Palsy, EEG- Electroencephalogram, F- Female, GTC- Generalised tonic-clonic, M- Male, MRI- Magnetic resonance imaging, NGT- nasogastric tube, NICU-Neonatal intensive care unit, VLCFA- Very long chain fatty acids, VUS- Variant of unknown significance.
