# Supplementary material for: Utility of next generation sequencing in paediatric neurological disorders: experience from South Africa
Source: Eur J Hum Genet. 2024 May 3;32(10):1314–8. doi: 10.1038/s41431-024-01582-2 (PMC11499987; doi:10.1038/s41431-024-01582-2)
Supplement: Supplementary file 6 — Supplementary Table 6 [file 41431_2024_1582_MOESM6_ESM.docx]

**Supplementary Table 6**

Identification of one pathogenic variant in a gene known to cause recessively-inherited disease

| Patient | Phenotypic features | Gene | OMIM | Inheritance/ zygosity | ACMG Classification |
| --- | --- | --- | --- | --- | --- |
| 8  (F) | Severe, early-onset epileptic encephalopathy. Left focal seizures, with "drop" attacks. Normal MRI brain. Abnormal EEG | *COG5*  NM_006348.3  c.2215C>T (p.Arg739*) | #613612 | AR / heterozygous | Pathogenic |
| 15(F) | Child with acute left hemiparesis, young stroke work-up normal except for MRI findings suggestive of leukoencephalopathy | *ACADS*  c.529T>C (p.Trp177Arg) | #606885 | AR /  heterozygous | Pathogenic |
| 47(F) | Congenital myopathy with progressive musculoskeletal complications and recurrent chest infections. | *TTN*  c.102234_102237del (p.Arg34079Serfs*9) | #188840 | AR /  heterozygous | Pathogenic |
| 75(M) | Neonatal seizures, requiring NGT feeding, Polymicrogyria on MRI, VLCFA indicative of peroxisomal disorder | *HSD17B4*  c.46G>A (p.Gly16Ser | #601860 | AR /  heterozygous | Pathogenic (inherited from the father) |
| 90(M) | 5 year old boy presents with 3 year history of spastic diplegia of unknown cause. | *BTD*  c.1330G>C (p.Asp444His) | #609019 | AR /  heterozygous | Pathogenic |
