# Supplementary material for: Utility of next generation sequencing in paediatric neurological disorders: experience from South Africa
Source: Eur J Hum Genet. 2024 May 3;32(10):1314–8. doi: 10.1038/s41431-024-01582-2 (PMC11499987; doi:10.1038/s41431-024-01582-2)
Supplement: Supplementary file 7 — Supplementary Table 7 [file 41431_2024_1582_MOESM7_ESM.docx]

**Supplementary table 7: Pathogenic variants identified incidentally, not considered to contribute to the phenotype**

| Patient | Phenotypic features | Gene | OMIM | Zygosity | Notes |
| --- | --- | --- | --- | --- | --- |
| 14  (M) | Seizures from 10month of age. Multiple seizure types, refractory to multiple ASM,  Microcephalic, happy demeanour and global developmental delay, non-verbal. | *UBE3A*  Deletion of entire coding sequence | #105830 | heterozygous | Parents need to be tested to verify whether this is significant – parent of origin effect |
| 17(M) | Congenital microcephaly and ventriculomegaly antenatally, developmental delay, spastic diplegia, no hyperbilirubinemia | *UGT1A1*  c.-41_-40dup  (Non-coding) | #191740 | heterozygous |  |
| 69(M) | Subacute onset of dense left hemiplegia. MRI features suggestive of adrenoleukodystrophy | *UGT1A1*  c.-41_-40dup  (Non-coding) | #191740 | homozygous |  |
| 70(M) | Weak, low Apgar score, contractures, ventilated for 15 days in NICU. | *CAPN3*  c.1466G>A (p.Arg489Gln) | #114240 | heterozygous |  |
| 96(F) | Profound bilateral Sensory-neural hearing loss and global developmental delay | *ATM*  c.2200dup (p.Val734Glyfs*4) | #607585 | heterozygous |  |
| 98(F) | Delayed walking and spastic paraplegia with normal Brain and spinal MRI | *NPHP1*  c.939+1G>T (Splice donor) | #607100 | heterozygous |  |
| 119(F) | Paroxysmal involuntary movements and dystonia with normal intellect | *BTD*  c.1330G>C (p.Asp444His)      *ADAR*  c.1786-1G>C (Splice acceptor)  *ADAR*  c.2082T>A (Silent) | #609019            #136920  #136920 | heterozygous        heterozygous  heterozygous | One ADAR pathogenic and one ADAR VUS – both inherited from the healthy mother |
