# Supplementary material for: Utility of next generation sequencing in paediatric neurological disorders: experience from South Africa
Source: Eur J Hum Genet. 2024 May 3;32(10):1314–8. doi: 10.1038/s41431-024-01582-2 (PMC11499987; doi:10.1038/s41431-024-01582-2)
Supplement: Supplementary file 8 — Supplementary Table 8 [file 41431_2024_1582_MOESM8_ESM.docx]

**Supplementary Table 8. Care-giver questionnaire answers.**

| **Did the result help explain the reason for your child illness?** | **Results of participants** |
| --- | --- |
| Yes | 25 (86%) |
| No | 3 (10%) |
| Some/little | 0 |
| Unknown | 1 (3%) |
| Not applicable | 0 |
| **Were any changes made to the treatment/ medication after the diagnosis?** |  |
| Yes | 14 (48%) |
| No | 11 (37%) |
| Some/little | 3 (10%) |
| Unknown | 1 (3%) |
| Not applicable | 0 |
| **Did the result help you to take better care for your child?** |  |
| Yes | 14 (48%) |
| No | 13 (44%) |
| Some/little | 2 (7%) |
| Unknown | 0 |
| Not applicable | 0 |
| **Did the results help you plan for future pregnancies?** |  |
| Yes | 8 (28%) |
| No | 5 (17%) |
| Some/little | 1 (3%) |
| Unknown | 3 (10%) |
| Not applicable | 12 (41%) |
| **Should prenatal testing become available, would you make use of it?** |  |
| Yes | 28 (97%) |
| No | 0 |
| Some/little | 0 |
| Unknown | 0 |
| Not applicable | 1 (3%) |
| **Did knowing the result bring you and your family any closure?** |  |
| Yes | 28 (97%) |
| No | 0 |
| Some/little | 1 (3%) |
| Unknown | 0 |
| Not applicable | 0 |
