# Supplementary material for: Utility of next generation sequencing in paediatric neurological disorders: experience from South Africa
Source: Eur J Hum Genet. 2024 May 3;32(10):1314–8. doi: 10.1038/s41431-024-01582-2 (PMC11499987; doi:10.1038/s41431-024-01582-2)
Supplement: Supplementary file 9 — Supplementary Table 9 [file 41431_2024_1582_MOESM9_ESM.docx]

**Supplementary Table 9. Comparison of NGS studies.**

| **Author** | **Neurological condition(s)** | **Type of study** | **Sample size** | **Number of genes per panel** | **Diagnostic yield** |
| --- | --- | --- | --- | --- | --- |
| Peng *et al*.(5)​ | Drug resistant epilepsy | Prospective | 273 | 540 genes | 31.5% |
| Graziola *et al*. ​(6 | Paediatric onset movement disorders | Retrospective | 204 | 102 genes | 28% |
| François-Heude *et al*.​(7) | Congenital muscular dystrophy | Retrospective | 28 | n=1- 18genes  n=12- 135 genes  n=15- 185 genes | 43% |
| Martinez-Granero *et al*. ​(8) | Neurodevelopmental delay | Retrospective | 1412 | 172 genes | 20% |
| Ko A *et al*.​(9) | Developmental/encephalopathic epilepsy | Prospective | 278 | 172 genes | 37.1% |
| Ganapathy *et al*.​(10) | Muscular dystrophy  Leukodystrophy  Ataxia | Prospective | 1012 | 4800 genes (TruSight One) | 64%  43%  43% |
| Ankala *et al*.​(11 | Neuromuscular disorders | Retrospective | - | 41 genes | 46% |
| Mergnac *et al*.​(12) | Metabolic conditions | Retrospective | 128 | 4811 genes (TRuSight One)  6699 genes (TruSight One expanded) | 39% |
| Antoniadi *et al*. ​(13) | Inherited peripheral neuropathy | Prospective | 448 | 56 genes | 31% |
| Sankaran *et al*. ​(14) | Leukodystrophy | Retrospective | 50 | 6440 genes | 60% |
| Gonzalez-Quereda​(15) | Neuromuscular disorders | Prospective | 207 | 116 genes | 49.3% |
| Essajee *et al*.​(4)  Akbar *et al*.(16) | Developmental and epileptic encephalopathy  Utility of genetic testing in pediatric epilepsy: Experience from a low to middle-income country | Retrospective  Retrospective | 41  77 | 300 genes  187genes | 48%  32% |
